# Supplementary material for: In Silico Molecular Studies of Antiophidic Properties of the Amazonian Tree Cordia nodosa Lam
Source: Molecules. 2019 Nov 16;24(22):4160. doi: 10.3390/molecules24224160 (PMC6891429; doi:10.3390/molecules24224160)
Supplement: Supplementary file 1 [file molecules-24-04160-s001.pdf]

Table S1. Similarity matrix for the target proteins. In each cell the upper number represents the percentage of identity and the lower the percentage of similarity for the protein in that row. The number in parenthesis represent the corresponding amino-acid number after Smith-Waterman comparison of the sequences. The cells are colored according to the percentage of similarity: <25% yellow, 25 – 50% orange, 50-75% green and >75% light blue. Diagonal cells with 100% identity are colored in dark blue. The protein IDs in the upper row have been colored by groups according to their similarity to the rest of the sequences.

	4GUE (305)	5A4W (212)	1QLL (121)	1XXS (122)	1Z76 (123)	2QOG (122)	2W12 (202)	3CXI (121)	3CYL (121)	3DSL (419)	4E0V (497)	5TFV (122)	5TS5 (484)	6CE2 (121)	6DIK (121)
4GUE (305)	100%	2.95% (9) 5.25% (16)	1.64% (5) 2.30% (7)	1.64 % (5) 2.95% (9)	1.97 % (6) 1.97 % (6)	1.31% (4) 1.31% (4)	8.20% (25) 14.10% (43)	1.97% (6) 2.95% (9)	1.64% (5) 2.30 % (7)	5.90% (18) 8.20% (25)	8.52% (26) 16.07 % (49)	1.97% (6) 3.28 % (10)	6.23% (19) 13.77% (42)	1.31 % (4) 1.64 % (5)	1.97 % (6) 2.95 % (9)
5A4W (212)	4.25% (9) 7.55% (16)	100	2.36% (5) 2.83% (6)	2.83% (6) 4.72% (10)	2.83% (6) 4.25 % (9)	3.77% (8) 4.25% (9)	6.60% (14) 9.91% (21)	2.36% (5) 2.83% (6)	2.62% (13) 3.43% (17)	4.25% (9) 8.49% (18)	19.81% (42) 35.38% (75)	2.36% (5) 2.83% (6)	19.81% (42) 35.38% (75)	5.19% (11) 6.60% (14)	2.36% (5) 2.83% (6)
1QLL (121)	4.13% (5) 5.79 % (7)	4.13 % (5) 4.96 % (6)	100%	93.39% (113) 95.04% (115)	48.76% (59) 70.25% (85)	48.76% (59) 62.81% (76)	4.96% (6) 7.44% (9)	98.35% (119) 99.17% (120)	99.17% (120) 99.17% (120)	14.05% (17) 21.49% (26)	19.83 % (24) 35.54 % (43)	60.33% (73) 71.07% (86)	5.79 % (7) 9.09 % (11)	86.78% (105) 89.26% (108)	99.17% (120) 99.17% (120)
1XXS (122)	4.10% (5) 7.38% (9)	4.92% (6) 8.20% (10)	92.62% (113) 94.26% (115)	100%	50.82% (62) 69.67% (85)	49.18% (60) 63.11% (77)	4.92% (6) 7.38% (9)	94.26% (115) 95.08% (116)	92.62% (113) 94.26% (115)	13.93% (17) 21.31% (26)	9.84% (12) 16.39% (20)	59.84% (73) 69.67% (85)	4.10% (5) 5.74% (7)	82.79% (101) 85.25% (104)	93.44% (114) 95.08% (116)
1Z76 (122)	4.92% (6) 4.92% (6)	4.92% (6) 7.38% (9)	48.36% (59) 69.67% (85)	50.82% (62) 69.67% (85)	100%	56.56% (69) 66.39% (81)	7.38% (9) 9.02% (11)	50.0 % (61) 70.49% (86)	48.36% (59) 69.67% (85)	18.85% (23) 28.69% (35)	8.20% (19) 9.02% (11)	56.56% (69) 71.31% (87)	8.20% (10) 9.84% (12)	50.00% (61) 65.57% (80)	49.18% (60) 70.49% (86)
2QOG (122)	3.28% (4) 3.28% (4)	6.56% (8) 7.38% (9)	48.36% (59) 62.30% (76)	49.18% (60) 63.11% (77)	56.56% (69) 66.39% (81)	100%	7.38% (9) 10.66% (13)	47.54% (58) 62.30% (76)	48.36% (59) 62.30% (76)	22.13% (27) 28.69% (35)	9.84% (12) 18.03% (22)	63.93% (78) 76.23% (93)	9.84% (12) 18.03% (22)	47.54% (58) 57.38% (70)	48.36% (59) 62.30% (76)
2W12 (202)	12.38% (25) 21. 29% (43)	6.93% (14) 10.40% (21)	2.97 % (6) 4.46 % (9)	2.97% (6) 4.46% (9)	4.46% (9) 5.45% (11)	4.46% (9) 6.44% (13)	100%	2.97% (6) 4.46% (9) 9	2.97% (6) 4.46% (9)	52.48% (106) 67.82% (137)	3.47% (7) 5.45% (11)	1.98% (4) 2.97% (6)	3.47% (7) 5.45% (11)	7.92% (16) 12.87% (26)	2.97% (6) 4.46 % (9)
3CXI (121)	4.96% (6) 7.44% (9)	4.13% (5) 4.96% (6)	98.35% (119) 99.17 % (120)	95.04% (115) 95.87% (116)	50.41% (61) 71.07% (86)	47.93% (58) 62.81% (76)	4.96% (6) 7.44% (9)	100%	98.35% (119) 99.17% (120)	14.05% (17) 21.49% (26)	19.83% (24) 34.71% (42))	59.50% (72) 70.25% (85)	5.79% (7) 9.09% (11)	85.95% (104) 89.26% (108)	99.17% (120) 100% (122)

3CYL (121)	4.13% (5) 5.79% (7)	10.74% (13) 14.05% (17t)	99.17% (120) 99.17% (120)	93.39% (113) 95.04% (115)	48.76% (59) 70.25% (85)	48.76% (59) 62.81 % (76)	4.96% (6) 7.44% (9)	98.35% (119) 99.17% (120)	100%	14.05% (17) 21.49% (26))	19.83% (24) 35.54% (43)	59.50% (72) 70.25% (85)	5.79% (7) 9.09% (11)	87.60% (106) 90.08% (109)	99.17% (120) 99.17% (120)
3DSL (479)	4.30% (18) 5.97% (25)	2.15% (9) 4.30% (18)	4.06% (17) 6.21% (26)	4.06% (26) 6.21% (26)	5.49% (23) 8.35% (35)	6.44% (35) 8.35% (35)	25.30% (106) 32.70% (137)	4.06% (17) 6.21% (26)	4.06% (17) 6.21% (26	100%	2.39% (10) 3.34 % (14)	4.06% (17) 5.97% (25)	2.39% (10) 3.58% (15)	4.06% (17) 6.68% (28)	4.06% (17) 6.21% (26)
4E0V (497)	5.23% (26) 9.86% (49)	8.45% (42) 15.09% (75)	4.83% (24) 8.65% (43)	2.41% (12) 4.02% (20)	2.01% (19) 2.21% (11)	2.41% (12) 4.43% (22)	1.41% (7) 2.21% (11)	4.83% (24) 8.45% (42	4.83% (24) 8.65% (43)	2.01% (10) 2.82 % (14)	100%	3.22% (16) 5.63% (28)	95.37% (474) 95.98% (477)	2.41% (12) 4.23% (21)	4.83% (24) 8.65% (43)
5TVF (122)	4.92% (6) 8.20% (10)	4.10% (5) 4.92% (6)	59.84% (73) 70.49% (86)	59.84% (73) 69.67% (85)	56.56% (69) 71.31% (87)	63.93% (78) 76.23% (93)	3.28% (4) 4.92% (6)	59.02% (72) 69.67% (85)	59.02% (72) 69.67% (85)	13.93% (17) 20.49% (25)	13.11% (16) 22.95% (28)	100%	13.11% (16) 22.95% (28)	56.56% (69) 66.39% (81)	59.02% (72) 69.67% (85)
5TS5 (484)	3.93% (19) 8.68% (42)	8.68% (42) 15.50% (75)	1.45 % (7) 2.27 % (11)	1.03% (5) 1.45% (7)	2.07% (10) 2.48% (12)	2.48% (12) 4.55% (22)	1.45% (7) 2.27% (11)	1.45 (7) 2.27% (11)	1.45 (7) 2.27% (11)	2.07% (10) 3.10% (15)	97.93% (474) 98.55% (477)	3.31% (16) 5.79% (28)	100%	2.48% (12) 4.34% (21)	1.45% (7) 2.27% (11)
6CE2 (121)	3.31% (4) 4.13% (5)	9.09% (11) 11.57% (14)	86.78% (105) 89.26% (108)	83.47% (101) 85.95% (104)	50.41% (61) 66.12% (80)	47.93% (58) 57.85% (70)	13.22% (16) 21.49% (26)	85.95% (104) 89.26% (108)	87.60% (106) 90.08% (109)	14.05% (17) 23.14% (28)	9.92% (12) 17.36% (21)	57.02% (69) 66.94% (81)	9.92% (12) 17.36% (21)	100%	86.78% (105) 89.26% (108)
6DIK (121)	4.96 % (6) 7.44 % (9)	4.13% (5) 4.96% (6)	99.17% (120) 99.17% (120)	94.21% (114) 95.87% (116)	49.59% (60) 71.07% (86)	48.76% (59) 62.81% (76)	4.96% (6) 7.44 % (9)	99.17% (120) 100% (122)	99.17% (120) 99.17% (120)	14.05% (17) 21.49% (26)	19.83% (43) 35.54% (43)	59.50% (72) 70.25% (85)	5.79% (7) 9.09% (11)	86.78% (105) 89.26% (108)	100%

Table S2. Heat map of sequence similarities as indicated in Table S1

	4GUE	5A4W	1QLL	1XXS	1Z76	2QOG	2W12	3CXI	3CYL	3DSL	4E0V	5TFV	5TS5	6CE2	6DIK
4GUE															
5A4W															
1QLL															
1XXS															
1Z76															
2QOG															
2W12															
3CXI															
3CYL															
3DSL															
4E0V															
5TVF															
5TS5															
6CE2															
6DIK															
